# Motivational theater to increase consumption of vegetable dishes by preschool children

**DOI:** 10.1186/s12966-017-0468-0

**Published:** 2017-02-07

**Authors:** Theresa Nicklas, Sandra Lopez, Yan Liu, Rabab Saab, Robert Reiher

**Affiliations:** 1 USDA/ARS Children’s Nutrition Research Center, Baylor College of Medicine, Houston, TX USA; 2 Founder of Esmartchoice, FutureWise Inc, and Innertainment, Burbank, CA USA

**Keywords:** Preschool children, Behavioral intervention, Vegetable intake, Motivational theater

## Abstract

**Background:**

By 3 years of age, many children have developed a dislike for certain foods, particularly vegetables. Seventy-five percent of young children consume less than the recommended levels for vegetables. The objective of this randomized feasibility intervention was to demonstrate the impact of an innovative approach to increase consumption of vegetable dishes by minority preschool children attending Head Start. The specific aims included the collection of data to assess feasibility and efficacy of the intervention.

**Methods:**

Both qualitative and quantitative assessments were conducted. Qualitative data was used for development of the intervention and for program feedback at post assessments. Two hundred fifty-three preschool children (49% boys; 66% Hispanics and 34% African-Americans; mean age 4.4 years) were randomized either to the intervention (*n* = 128) or the control group (*n* = 125). The teacher/parent intervention group showed the children videotaped (DVD) puppet shows. Based on the theoretical framework “transportation into a narrative world”, three professionally developed characters, unique storylines and an engaging, repetitious song were incorporated in four 20-min DVD puppet shows. Prior to lunch each show was shown for five consecutive days in school and a minimum of once in the home. Digital photography was used in school to assess consumption of vegetable dishes at the lunch meal (quantitative assessment). At home parents were asked to complete the booklet questions corresponding to each DVD; questions could be answered correctly only if parents watched the DVD with their child. A multilevel mixed-effect model was used to analyze the data, adjusting for age, gender, and ethnicity.

**Results:**

Children in the intervention group significantly (*p* < 0.0001) increased consumption of vegetable dishes from baseline to follow-up compared to no change in the control group. At follow-up, the intervention group continued to have significantly (*p* = 0.022) higher intake of vegetable dishes compared to the control group. Sixty percent of the mothers completed the booklet’s questions with 76 to 98% correct responses.

**Conclusion:**

Using theory-based motivational theater with multiple exposures may be an effective behavioral intervention to increase consumption of vegetable dishes by preschool children that can be easily disseminated to a large sample.

**Trial Registration:**

ClinicalTrials.gov; Identifier: NCT02216968

## Background

Higher vegetable intake is associated with a lower risk of diabetes [1], cardiovascular disease [2, 3], several cancers [4–6], and obesity [7–10]. Dietary behaviors initiated in early childhood may persist into adulthood [11, 12], lowering the risk of chronic diseases. By 3 years of age, many children have developed a dislike for certain foods, particularly vegetables [13–16]. Seventy-five percent of young children consumed less than the recommended levels for vegetables, with median intakes less than one cup per day [17]. French fries constituted about 23% of all vegetables consumed. Many preschool children are reluctant not only to eat vegetables, but even to taste vegetables [18–21], which is reflected in the very low intake of vegetables observed in this age group [16, 22–25]. This research focused on increasing consumption of vegetable dishes in two low-income minority groups, African-American (AA) and Hispanic-American (HA), that have disproportionately higher risk for developing obesity [26, 27] and cancers [28] later in life. Since children’s food preferences and practices are initiated early in life (e.g. 2- to 5-year-olds) [29–34], early dietary intervention programs will have immediate nutritional benefit for young children, and should reduce chronic disease risk when these learned habits are carried into the adult years [35–37].

Health education approaches involving drama or theatre performances in the school setting are underused [38–41]. Theatre performances are a promising approach for engaging children with messages about healthy eating [42] and weight related behaviors [41]. One study used theatre to change food-related knowledge and food choices of vegetables among children in grades 1–6 [42]. The theatre intervention made a substantial impact on children’s reported food choices, but unfortunately the study design lacked a control group. Theatre interventions appear to be most effective when they involve a story (also called a narrative). A narrative’s unique immersive capacity engenders arousal and attention [43]; helps create a deep affection for the characters [44, 45]; and absorbs the viewers in an immersive fictional world [46]. Compared to live puppet shows, videotaped (DVD) puppet shows are relatively inexpensive to reproduce and can be shown to a wider audience numerous times in a variety of settings. Ninety-seven percent of US households have at least one DVD player [47, 48].

Several behavioral strategies have been used to increase vegetable intake in preschool children [49–52]. The one strategy most relevant to this intervention was the incorporation of vegetables into mixed dishes which is also one of the tips cited in USDA’s ChooseMyPlate.gov [53]. Approximately 59% of total vegetable intake among children and adolescents come from whole forms of vegetables with 41% coming from a mixed dish [54]. This is reflected in the Houston Head Start (HS) menus. Thus, the focus of the intervention was to increase preschool children’s consumption of dishes containing vegetables.

This study was a dietary change intervention targeted at children in HS. The goal of this study was to test the feasibility of an innovative approach to increase the consumption of vegetable dishes by preschool children who were predominantly low-income African Americans (AA) and Hispanics. The hypothesis was that the intervention with a parent/teacher component would increase consumption of vegetable dishes by preschool children.

### General Procedures Phase I

#### Qualitative Assessment (Program Development)

Qualitative assessment was the primary focus of Phase I for obtaining information that was used for development of the characters and storylines for the Puppet shows. Phase I was conducted at six HS centers in three districts. In phase I, 30 teachers (50% AA and 50% Hispanics), and 30 parents (50% AA and 50% Hispanics), were recruited to participate in audio recorded, semi-structured, individual interviews to obtain their opinions about healthy eating/nonhealthy eating, media, cooking, and to obtain feedback, specifically addressing cultural and ethnic differences, which was used for development of the characters and storylines for the puppet shows. Teachers and parents were given the list of questions to take home and prepare for the interview, and parents were provided two additional questionnaires and were asked to prepare a list of their child’s favorite foods. The information collected was used to develop four puppet shows to be used in the intervention study in the fall of 2014. The interviews were conducted in a semiprivate area at participating HS centers.

#### Development of Characters and Storylines

Characters and storylines were the main components of the narrative and were important determinants of its immersive quality.

##### Characters

**Fig. 1:**
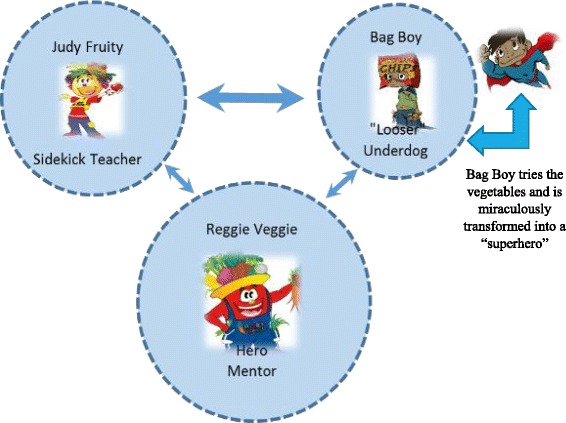
Character Dynamics. Footnotes: Size of circles = Importance of Character. Arrows = communication between characters. Width of arrows = Strength of relationship

Three characters were professionally developed: Reggie Veggie, Judy Fruity, and Bag Boy. Character development required a rather rigorous process which involved character design, development, personality profile, and character dynamic. Reggie Veggie was a high-energy, humorous, and slightly bubbly hero/creator/warrior and a strong mentor to Judy Fruity and Bag Boy (Fig. 1). Judy Fruity was intelligent, clever, and a teacher portrayed as a “sleeper” sidekick supporting Reggie Veggie in getting children to eat vegetables and to change Bag Boy and make him healthy. However, Bag Boy was the “wanna be loser” underdog who refused to eat vegetables and wanted only chips and candy. But Reggie Veggie persuaded Bag Boy, with the encouragement/support of Judy Fruity, on the importance of eating vegetables and the very special properties and nutritional value of vegetables. Bag Boy then tries the vegetables and is miraculously transformed into a “superhero” (Fig. 1).

##### Storyline

Each puppet show had a story to tell with a repetitious, engaging song. The children learned the song, danced and jumped around, and sang the song during the shows and even throughout the day, including at home. The repetitious theme was that eating vegetables “Give You the Power to Play”. Four behavioral strategies were incorporated into each puppet show: role modeling, reinforcement, encouragement, and rationale/reason. Puppet show one focused on vegetables that “crunched” (i.e., carrots, sweet potatoes) and were a good source of vitamins A and C. Puppet show two focused on vegetables that “Pop and Roll” (i.e., cherry tomatoes and peas) and were a good source of lycopene or B vitamins. Puppet show three focused on vegetables that were “Cool Veggies” (i.e., green beans, lettuce) and were a good source of vitamins C and K. Puppet show four focused on vegetables that were used for a “Craft Day” (i.e., broccoli, squash) and were a good source of calcium or vitamins A and C. The puppet shows were available in both English and Spanish.

## Methods

### Theoretical Framework

This feasibility study explored an intervention designed to motivate and persuade children to make healthful vegetable choices by reaching them in a language they understood and in ways that were engaging and entertaining. The intervention included four DVDs (videos) theater-based puppet shows that aimed at persuading children to increase vegetable consumption through encouragement, rationale/reason, reinforcement, and role modeling. The theoretical basis for the puppet intervention was “transportation into a narrative world” [55–58], which has been identified as a mechanism of narrative impact. The impact of narrative messages on children’s attitudes is dependent on the extent to which they become involved with the narrative [59]. Researchers have discussed this concept of absorption into a narrative [55–58]. This phenomenon of transportation is a convergent process where all mental systems and capacities become focused on the events in the narrative. Transportation is an integrative melding of attention, imagery, and feelings, focused on story events [56–58] which influence the audience’s cognitive, affective, and potentially, health behaviors. A person that has been “transported” suspends normal assumptions and enters the narrative as a new immersive frame of reference [60]. Transportation promotes the suspension of disbelief and the reduction of counterarguments, enables the story experience to become a personal experience, and creates player’s deep affection for narrative protagonists. As one’s thoughts center on the story, they respond emotionally to the characters and events and picture the events as they unfold. As a learning process “transportation into a narrative world” [56] reflects the shift or transfer of an individual in both the cognitive and emotional domain. The major dimensions of transportation [56] include cognitive attention to the story; emotional involvement; feelings of suspense; a lack of awareness of the surroundings; and, existence of mental imagery. Transportation complements the behavioral change theory, Social Cognitive Theory [61] in that the intervention showed role-modeling, encouragement, rationale/reason, and reinforcement for consuming vegetables. Briefly, the audience becomes immersed in a story, followed by character modeling of vegetable consumption and virtual tasting of vegetables, with increased vegetable preference leading to increased vegetable consumption.

#### General Procedures Phase II

Phase II was to test the feasibility of this innovative approach to increase the amount of vegetable dishes consumed by preschool children who were predominantly low-income AA and Hispanics. A secondary aim of Phase II was to obtain program feedback.

#### Recruitment Strategy

The study was approved by Baylor College of Medicine (BCM) Institutional Review Board, and consent forms were signed before the initiation of observation. Recruitment strategies included flyers that were sent to the home with the children, presentations at parent meetings, face-to-face recruitment during child drop-off and pickup at HS, and active involvement of the HS manager and staff in the recruitment process. These strategies were used in all recruitment phases of this study. Flyers that briefly explained the study activities and requested parent and child contact information were sent home with all children at each of the six participating HS centers. The study coordinator collected the completed flyers that were returned to the school administration. When less than 50% of the schools’ enrollment did not return flyers, arrangements were made to meet parents to provide a brief description of the study activities, to encourage enrollment, and to arrange for drop off or pick up of consent forms.

#### Intervention

The intervention was conducted in 11 classrooms at three schools in three HS districts for four consecutive weeks to accommodate the series of four puppet shows. Based on the theoretical framework “transportation into a narrative world”, three professionally developed characters, unique storylines and an engaging, repetitious song were incorporated in four 20-min videotaped puppet shows. Prior to lunch each show was shown for five consecutive days in HS and a minimum of once in the home. On Mondays each intervention child took home a bag including the DVD video for that week, a pamphlet, main ingredients to prepare a simple vegetable snack, crayons, and a disposable camera (if parents did not have a smart phone) to use as instructed in the booklets. The pamphlet materials included positive feeding practices, instructions on snack recipe preparation, content information contained in the videos, in-home instructions on taking and sending pictures of the children preparing and tasting the snack, and questions for parents about the video content. The questions on the video content could be answered correctly only if the parent had watched the video. On Mondays of weeks two, three, four, and five, researchers gathered from the teachers the booklets that were returned by the parents from the previous week. As an incentive, each time a family returned a completed pamphlet, they were entered into a drawing to have a professionally taken family portrait.

#### Qualitative Assessment (Program Feedback)

Four focus groups (FGs) were conducted with parents (*n* = 19) who participated in the intervention were conducted at three HS centers in three different HS districts. The focus groups (FGs) were moderated by a trained coordinator and in the presence of a research intern who was involved in conducting the study. The FGs were audiotaped, and participants were compensated $25 cash for their time. Refreshments and childcare were provided at each FG. Reading and following the instructions in each booklet, watching the videos, and preparing a vegetable snack were the major components of the in-home intervention.

A total of three FGs were conducted with teachers (*n* = 22) and, they were audiotaped, and participants were compensated $25 cash for their time. Refreshments were provided during the FGs. The moderator used a written guide to assist in getting enough feedback from the teachers concerning the implementation of the intervention at HS, any changes they thought would make the program more successful, and strategies for optimizing compliance of the participating families with the take-home portion of the intervention. They were asked specifically about children’s reactions to the characters, video content, and what they thought the children learned from watching the videos.

#### Quantitative Assessment

The digital photography method was used to assess consumption of vegetable dishes in children [62, 63]. Assessment was done 2 days prior to the 4-week intervention and 2 days after the intervention was completed. Trained assessors used digital cameras to capture images of the vegetable dishes for initial serving, before additional servings, and after additional servings of vegetable dishes. Each assessor documented the picture numbers corresponding to each child being observed on the meal observation form (MOF). Placement mats with children’s identification numbers were used. As the children finished eating, plates were marked with the corresponding personal identification number and then transferred to the plate waste station for weighing. Plate waste measurement in grams (g) of each vegetable dish per child was recorded on the MOF to identify individual consumption of vegetable dishes (amount served minus plate waste). Interrater measurements were done on more than 10% of the sample. During the 4-week intervention period the control group did not receive any alternate intervention. Prior to lunch, the intervention teachers/aides were encouraged to introduce the lunch menu items to the children and to emphasize the vegetables being served and in what dish. The school lunch menus were not altered to include more whole forms of vegetables or to increase the amount served. The intent was to increase the awareness of the children of what vegetables were being served and in what dishes. It was not possible to observe the control teachers to see if they introduced the vegetables dishes to the children; however, they were not encouraged to do so. The three ways vegetables were served in HS were in entrée vegetable dishes, vegetable mixed dish, or as a whole vegetable dish.

#### Analytical procedures

All analyses were conducted using Statistical Analysis Software (SAS) [64]. Significance was set at *p* < 0.05. Numerical (Skewness, Kurtosis, and Kolmogorov-Smirnov D) and graphical methods were used to test for data normality. Baseline demographic characteristics were examined for differences between groups by using a chi-square analysis for the categorical variables and an analysis of variance (ANOVA) for continuous variables, respectively. Intent-to-treat analysis assessed the influence of dropouts on the effect of the intervention).

The hypothesis was that children who participated in the intervention would be significantly more likely to consume more, or at least taste more, dishes with vegetables than the control group. The multilevel mixed-effect model was used to account for the clustering of individuals within schools. This mixed model technique also allowed for unequal numbers of participants at baseline and follow-up. The model included group and time as fixed effects and schools as a random effect. The interaction term for group (intervention or control) by time (pre-test and post-test) was examined to determine whether the change in the intervention group from pre-test to post-test was significantly different from the change in the control group over time. The model was adjusted for child’s gender, age, and ethnicity to account for the confounding influence. Standardized effect sizes were computed according to Cohen [55]. Small, medium, and large effect size is defined as 0.2, 0.5, and 0.8, respectively.

## Results

### Demographic Characteristics (Table 1)

**Table 1:** General demographic characteristics of subjects at baseline

	Total	Control	Intervention	*P*-value
Sample, *n* (%)	253 (100.00)	125 (49.41)	128 (50.59)	
Gender, *n* (%)				0.658
Boys	125 (49.41)	60 (48.00)	65 (50.78)	
Girls	128 (50.59)	65 (52.00)	63 (49.22)	
Race/Ethnicity, *n* (%)				0.065
Hispanics	166 (65.61)	89 (53.61)	77 (46.39)	
African American	87 (34.39)	36 (41.38)	51 (58.62)	
Age, $\overline{\mathrm{x}}$ ± SD	4.43 ± 0.65	4.38 ± 0.69	4.47 ± 0.54	0.261

$\overline{\mathrm{x}}$ = mean, *SD* standard deviation

**Fig. 2:**
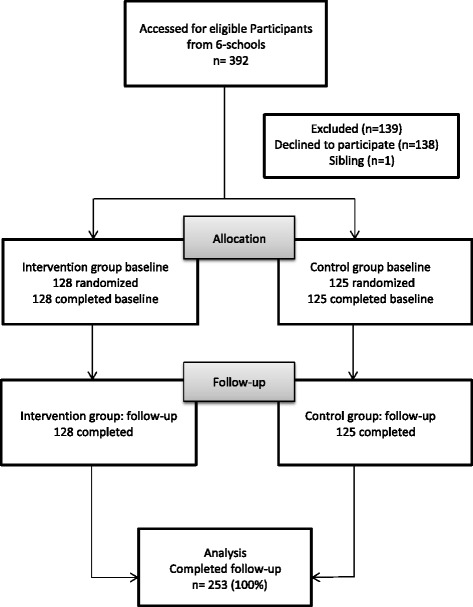
Flowchart of Final Sample Selection

A total of 253 preschool children (mean age = 4.4 years) participated in the study (128 intervention, 125 control,). The sample was 49% boys and 66% Hispanics and 34% AA. There were no significant differences in demographic characteristics between the control and intervention groups. Figure 2 depicts The CONSORT diagram for the Intent-to-treat analysis result.

### Changes in Vegetable Consumption (Fig. 3)

**Fig. 3:**
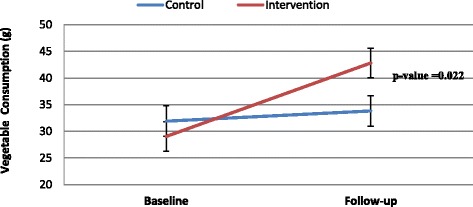
Increased Consumption of Vegetable Dishes After Intervention. Footnotes: Within group: Intervention group had increased consumption of vegetable dishes from baseline to follow-up (*p* < 0.0001). Between group: at follow-up, Intervention group had significantly higher consumption of vegetables dishes than the control group at follow-up (*p* = 0.022)

Children in the intervention group significantly (*p* < 0.0001) increased consumption of vegetable dishes from baseline to follow-up compared to no change in the control group. At follow-up, the intervention group continued to have significantly (*p* = 0.022) higher intake of vegetable dishes compared to the control group. The intervention effect size was considered small (Cohen’s d = 0.28). Sixty percent of the mothers completed the questions in the booklets, with 76 to 98% correct responses depending on which video was watched (Table 2).

**Table 2:** Evaluation results of the parent component^a^

	Response	Number	Percent
Videotape 1 (Crunch) Questions			
Name a vitamin that carrots are an excellent source of?	Correct	48	98.0
	Not correct	1	2.0
Which disease can eating carrots help prevent?	Correct	41	87.2
	Not correct	6	12.8
Sweet potatoes are good for which body organ?	Correct	35	76.1
	Not correct	11	23.9
What was 1 of the 2 highlighted vegetables in the video?	Correct	44	95.7
	Not correct	2	4.4
Videotape 2 (Pop and Roll) Questions			
Name something that cherry tomatoes have in them?	Correct	44	88.0
	Not correct	6	12.0
Name a vitamin that peas have?	Correct	42	87.5
	Not correct	6	12.5
What was 1 of the 2 highlighted vegetables in the video?	Correct	43	97.7
	Not correct	1	2.3
Videotape 3 (Cool Veggies) Questions			
Name a nutrient that green beans have?	Correct	27	84.4
	Not correct	5	15.6
What nutritional benefit does romaine lettuce have on the body?	Correct	3	9.4
	Not correct	29	90.6
What was 1 of the 2 highlighted vegetables in the video?	Correct	30	96.8
	Not correct	1	3.2
Videotape 4 (Craft Day) Questions			
Name a nutrient in broccoli?	Correct	28	93.3
	Not correct	2	6.7
Name a mineral that squash has?	Correct	27	90.0
	Not correct	3	10.0
What was 1 of the 2 highlighted vegetables in the video?	Correct	26	96.3
	Not correct	1	3.7

^a^Approximately 79% Response Rate

### Program Feedback From Parents

#### Videos

When parents were asked about how often their children viewed the videos 100% responded their child viewed at least three of the puppet videos. Parents agreed that watching the videos helped children understand that eating vegetables would make them strong and healthy. Children learned that vegetables provided “vitamins” and energy, and they also could identify junk food, and some children repeated that eating junk food made them tired. Some mothers said that watching the videos educated them on the health benefits of the featured vegetables and that they were motivated to cook, eat, and serve more of the vegetables shown in the videos to their families.

All parents reported their children requested watching the videos over and over again; 75–90% of parents reported watching the videos at least once. Of those who did not watch all of the videos, 10–25% reported watching only parts. One or two of the parents who participated in the focus group reported not watching the videos because they were too busy with other children, school work, and household chores. A suggestion was made to have a parent–child meeting at schools once a week to watch the videos together. A mom reported that interacting with her child while cooking helped her child eat more vegetables. Parents said the intervention helped them learn new and fun ways of preparing and eating vegetables. They liked the positivity and energy of the characters, who they believed helped and encouraged the children to try new vegetables. The parents learned the importance of eating vegetables and its impact on general health; Most of the parents agreed that the videos accomplished the goal of getting children to eat more vegetables. All of the parents rated the message to eat more vegetables to be very important, and most said they would purchase these videos in stores if they saw them at a reasonable price.

Parents also reported that their children liked the song and the names of the characters. Parents believed the reason their children liked the videos was because it was cartoon-like, the characters interacted with the audience (children), and encouraged them to participate because of the upbeat singing and dancing and the bright colors. Children jumped with excitement and joy as the characters appeared on the screen. Some of the children loved Reggie, some especially the girls loved Judy, and some loved Bag Boy. Some of the children liked Bag Boy because they could associate with him the most because they, like Bag Boy, didn’t want to eat vegetables. According to the parents, Bag Boy was successful in getting the children to taste the vegetables. The children would mimic the characters; importantly, the parents said their children noticed and commented on how the characters were so nice and polite to each other in the videos. Parents thought the display of the importance of friendship was a great attribute of the videos. The parents reported the children had strong positive reactions to the song and they memorized it quickly. They continued to sing the song even though the intervention had been completed for months.

#### Booklets

Some parents thought the booklets were a way to inform them and their children about vegetables. Parents confirmed that the questions in the booklets were simple and filling them out was informative. They all clarified that their children enjoyed coloring the vegetables and characters in the booklets. The instructions on taking and sending pictures were clear, although some parents suggested dedicating a live session for instructions on taking and sending the pictures. There were just a few issues with kids not wanting to wait till after the picture was taken to start eating. A suggestion was also made to send text messages as reminders to take the pictures and to send them. Some parents complained that their busy schedule was a hindrance to filling out and turning in the booklets.

#### Snack preparation

Most parents reported they prepared the snacks, but a few reported not preparing certain ones knowing their child would not like eating it. Most mothers commented that snack preparation created more interaction and bonding with their child; children enjoyed helping in the preparation of the vegetables because it made them feel “big”. After watching the videos the children were more willing to try vegetables, especially when they helped in the preparation of the recipe snack. Though some of the parents did not have time or didn’t make the time to be involved in their children’s activities, overall, the parents thought the study was good as is, the videos were not long, the recipes were not complicated, and this was a good way to keep parents involved and informed.

### Program Feedback From Teachers

All the teachers reported that their students watched all of the videos for most of the duration during class time. In their opinion, some of the reasons that kept students from watching the entire video were hyperactivity, absenteeism, and a few behavior issues. They suggested technical solutions that could improve on gaining the attention of the children which included projecting the shows on a larger screen to improve visibility and using better-quality speakers or sound system. Another suggestion was incorporating more singing throughout the shows to maximize the attention and involvement of the children.

Teachers reported that the children liked the videos because of the song; they sang and danced along. Most children remembered the song in such a way that they started singing it before the video was played and they would continue with the song throughout the day. Children hummed the song at naptime and then sang it all together. They liked how the characters danced, the sound effects, and the colorful vegetables in the videos. Teachers believed that some children identified with Reggie; they called out his name and sang along with him. Other teachers reported that children identified with Bag Boy; they wanted a chip bag to put on their head just like Bag Boy. Similar to the children, Bag Boy did not like vegetables but his appreciation for vegetables changed after tasting them. Every time kids refused to eat their vegetables, teachers encouraged them to do so using Bag Boy as a model. Teachers thought some children didn’t identify with Judy Fruity because they viewed her as a teacher to Bag Boy. Teachers reported that after viewing the videos children started liking carrots, spinach, peas, and sugar snap peas. The concept of having energy as a result of eating vegetables was quite appealing to the children, so much so they would remind each other saying “You’re not eating your vegetables you won’t have energy.” The children asked if eating peas, carrots, or green beans gave them the “Power to Play”. Children shouted “Eat’em Up” (the final encouraging message at the end of each video) at lunch every time vegetables were served. Teachers reported that the intervention had a positive impact on children; those children who ate their vegetables were proud of themselves and encouraged their peers to do the same. Viewing the videotaped puppet shows made the children brave and encouraged them to taste the vegetables they avoided eating, especially the green ones. Importantly, teachers reported that after viewing the videos more children requested seconds of vegetables instead of entrée or starch because vegetables provided them with energy.

Children learned the names and shapes of different vegetables. They also went so far as to identify the vegetable in the video as a “crunch” or a “pop” vegetable. Teachers also noticed that the children learned from viewing the videos that eating vegetables makes one grow to be healthy; why vegetables were important; what vegetables do to the body; social/emotional skills; and the sounds of the vegetables. To improve the program, teachers suggested a few changes such as: preparing snacks in front of the parents; having more demonstrations of the activities included in the booklets; having some of the characters dress up and visit with the children at HS; and having a live puppet show with the actual vegetables which would provide children with the opportunity to explore all five senses. Other suggestions were to create an app or web link to access and watch the videos.

To summarize, teachers understood that the purpose of the intervention was getting the children to eat more vegetables to be healthier. They thought the videos were a great idea to promote vegetable consumption and teach the children new vocabulary. Teachers expressed that the videos had an effect not only on the children but also on the teachers. Watching the videos made them aware that in order to get their children to eat vegetables, role models (teachers and parents) need to be better educated on vegetables and they need to eat them as well.

## Discussion

This pilot feasibility study was a dietary change intervention targeted at children in HS. The goal of this exploratory study was to test an innovation approach to increasing intake of vegetable dishes among minority preschool children. The intervention was motivational theatre performances designed to engage children to eat a variety of vegetables by reaching them in a language they speak and in ways that are engaging and entertaining. Theatre interventions appear to be most effective when they involve a story (also called a narrative). A narrative’s unique immersive capacity engenders arousal and attention, helps create a deep affection for the characters, and absorbs the viewers in an immersive fictional world. Compared to live puppet shows, videotaped puppet shows are relatively inexpensive to reproduce and can be shown to a wider audience, numerous times, and in a variety of settings.

This study provides data supporting the use of videotaped puppet shows using a storyline, a repetitious song, and three well-tested characters with their own personality profiles to change eating behavior. The videotaped puppet shows, shown on repeated occasions, significantly increased consumption of vegetable dishes by preschool children. The present results add to the body of literature showing that multiple exposures to a theatre intervention with a strong theoretical framework can have a significant impact on young children’s consumption of vegetable dishes. The use of puppet shows is an appropriate medium of intervention to use with preschool children based on their level of cognitive development. Children of this age are preliterate. Therefore, they are almost entirely visual and attracted to visual media. Puppet shows are an example of a potent visual medium. Additional research is needed to determine whether the positive gains from theatrical interventions targeting preschool children, with a sound theoretical framework, and observed short-term, are sustained over time. Although the effect size was small, the increase in consumption of vegetable dishes was significant and supports feasibility.

## Conclusion

Using theory-based motivational theater with multiple exposures and related activities may be an effective behavioral intervention to increase consumption of vegetable dishes by preschool children that can be easily disseminated to a large sample.

## Abbreviations

AA: African American
AIDS: Acquired immunodeficiency syndrome
ANOVA: Analysis of variance
BCM: Baylor College of Medicine
DVD: Digital versatile/video disc
FG: Focus group
HIV: Human immunodeficiency virus
HS: Head start
MOF: Meal observation form
USDA: United States Department of Agriculture
VCR: Videocassette recorder
